# Parabolic flight induces site specific microbiome changes in women

**DOI:** 10.3389/fmicb.2026.1817099

**Published:** 2026-05-25

**Authors:** Begum Aydogan Mathyk, Rohit Shukla, Vivek Kumar, Sidharth P. Mishra, Shawna Pandya, Norah Patten, Kellie Gerardi, Heather Wright Beatty, Aaron H. Persad, Anthony N. Imudia, Hariom Yadav, Shalini Jain

**Affiliations:** 1 Division of Reproductive Endocrinology and Infertility, Department of Obstetrics and Gynecology, University of South Florida Morsani College of Medicine, Tampa, FL, United States; 2 Aerospace Science, Technology, Research and Applications (ASTRA) Center, Department of Mechanical and Aerospace Engineering, University of South Florida, Tampa, FL, United States; 3 Department of Neurosurgery, Brain and Spine, University of South Florida, Tampa, FL, United States; 4 USF Center for Microbiome Research, Microbiomes Institute, Tampa, FL, United States; 5 International Institute for Astronautical Sciences, Boulder, CO, United States; 6 Faculty of Medicine & Dentistry, University of Alberta, Edmonton, AB, Canada; 7 Flight Research Laboratory, Aerospace, National Research Council Canada, Ottawa, ON, Canada; 8 Department of Engineering, University of Maryland Eastern Shore, Princess Anne, MD, United States; 9 Shady Grove Fertility, Tampa, FL, United States

**Keywords:** *Lactobacillus*, parabolic flight, space medicine, spaceflight, vaginal microbiome

## Abstract

**Introduction:**

The vaginal microbiome plays a central role in women’s health by supporting immune function, maintaining mucosal homeostasis, and preventing infections. Spaceflight and its analogs can induce acute physiological stress, which can alter host microbiome interactions. While other studies have analyzed the microbiome changes at certain body sites, the female-specific microbiome changes have not been explored in depth in space medicine research.

**Methods:**

Pre- and post-parabolic flight vaginal and oral microbiome were analyzed via metagenomic shotgun sequencing to assess taxonomic composition and metabolic pathways. Host DNA and bad quality sequences were removed using the KneadData tool. Taxonomic and functional profiles were analyzed with MetaPhlAn and HUMAnN. Microbiome data were integrated with stress response parameters including cortisol, proinflammatory cytokines, and urinary short-chain fatty acids.

**Results:**

Both alpha- and beta diversity analysis showed minimal impact of parabolic flight on oral microbiome while vaginal microbiome showed significant differences. Taxonomic profiling showed marked restructuring of the vaginal microbiome, characterized by increased *Firmicutes* dominance and enrichment of *Lactobacillus* species, particularly *Lactobacillus crispatus* and *Lactobacillus jensenii*, whereas oral microbiome stayed relatively stable. Overall, only 2.54% of oral species showed significant postflight changes compared to 57.9% of vaginal species (*p* < 0.0001). Random Forest model identified *L. crispatus* as a key discriminator of postflight vaginal microbiome composition. Metabolic pathway analysis revealed minimal postflight pathway redistribution in saliva samples but greater number of changes in the vaginal microbiome, with significant postflight enrichment of fatty acid biosynthesis and nucleotide metabolism. Vaginal samples demonstrated a threefold greater proportion of altered metabolic pathways compared to oral samples. In addition, urinary acetate, butyrate, and valeric acid levels were significantly reduced postflight. Salivary cortisol increased postflight and positively correlated with *L. jensenii*.

**Conclusion:**

Parabolic flight induces body site-specific microbiome changes in reproductive age women, with greater taxonomic and functional metabolic remodeling in the vaginal microbiome than in the oral microbiome. These findings highlight the sensitivity of the vaginal microbial ecosystem to spaceflight stressors and underscore the need for longitudinal and mechanistic studies to determine the persistence, clinical significance, and potential health implications of these changes during longer duration space missions.

## Graphical abstract

**Figure:**
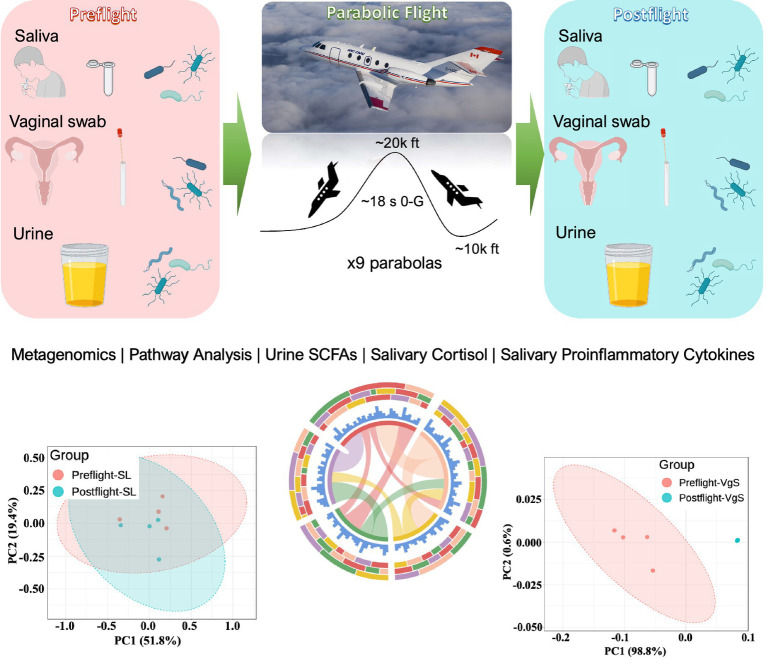

## Introduction

As human space exploration moves toward longer duration missions, including lunar habitation and deep space travel, and a more global representation of the population, understanding the biological consequences of spaceflight on astronaut health has become increasingly important. Although women now represent a growing proportion of the astronaut corps, space medicine research has historically underrepresented female-specific systems, leaving critical knowledge gaps in women’s health during spaceflight (Mathyk et al., 2024).

The vaginal microbiome is a key determinant of female reproductive and urogenital health, contributing to mucosal immunity, epithelial barrier maintenance, and protection against pathogenic infection. In reproductive age women, the vaginal microbiome is commonly dominated by *Lactobacillus* species, which maintain an acidic vaginal environment through the production of lactic acid and other antimicrobial metabolites. Disruption of this microbial balance has been associated with inflammation, increased infection susceptibility, and altered immune responses. Over the past decades, the clinical relevance of the vaginal microbiome has extended beyond its traditional associations with bacterial vaginosis or vulvovaginal candidiasis, emerging as a non-invasive biomarker for gynecological disease detection, pregnancy, and health monitoring (Cheng et al., 2025; Black et al., 2026). For instance, in endometriosis, microbial dysbiosis has been associated with chronic inflammation, altered immune responses, and disease progression (MacSharry et al., 2024).

Spaceflight associated stressors, including microgravity, radiation exposure, circadian disruption, and psychological stress have been shown to modulate host immunity and microbial behavior (Winer et al., 2025). Microgravity alters epithelial physiology, fluid dynamics, and immune signaling, while also influencing microbial growth, gene expression, and virulence factors. Previous studies have demonstrated spaceflight-induced changes in the gut, skin, and oral microbiomes; however, the effects of spaceflight conditions on the vaginal microbiome remain largely unexplored. Parabolic flight offers a validated terrestrial analog for studying the gravitational changes, characterized by repetitive transitions between hypergravity and microgravity that can elicit changes in host and microbial responses. Prior studies reported that gravitational changes impact immune cell and endothelial interactions after parabolic flight (Du et al., 2025). Characterizing how altered gravity exposure influences the vaginal microbiome is particularly relevant for female astronauts, as vaginal dysbiosis may compromise mucosal homeostasis and immune defense and may lead to increased incidence of genitourinary pathologies in an environment where medical resources are limited. As a longer duration of spaceflight is the goal, temporary and permanent alterations in the microbiome impacting health need to be investigated in astronauts.

Understanding how exposure to altered gravitational environments impacts the vaginal microbiome, even with brief exposures, can further inform our understanding of vaginal health in spaceflight. In this study, we investigate the impact of parabolic flight in the oral and vaginal microbiomes of reproductive age women using metagenomics shotgun sequencing to assess taxonomic composition and functional potential. We aim to provide novel insights into microbial adaptation under altered gravitational conditions and highlight the importance of incorporating female-specific microbiome research into space medicine.

## Materials and methods

Following institutional ethics approval, four reproductive age women provided written informed consent prior to the parabolic flight. All participants (*n* = 4) were healthy at the time of enrollment and had no reported gynecological infections or recent antibiotic or probiotic supplement use prior to sample collection. The age range of the participants was 35–42 years (mean ± SD: 39.25 ± 2.98), and BMI ranged from 17.9–25.5 kg/m^2^ (mean ± SD: 20.9 ± 3.23). Saliva and vaginal sample collection kits were provided, which included gloves, saliva collection tubes, swabs, and preservatives. For saliva collection, participants were instructed not to eat, chew, or drink for at least 2 h prior. Saliva samples were aliquoted for oral microbiome, cortisol, and inflammatory cytokine panel analysis. Whole saliva (5 mL) was collected in a 50 mL tube and then equally aliquoted into four separate 2 mL tubes for different purposes before being immediately stored at −80 °C. Unstimulated saliva samples were collected using the passive drool method to minimize external stimulation and variability. Sterile swabs were used for vaginal sample collection. Detailed instructions, along with representative images, were provided to the participants, along with instructions to strictly avoid vulvar skin contact to reduce contamination from external microbiota. Baseline preflight and postflight samples were collected at the same time from all participants. Sample size was eight for each parameter, and all samples were stored at −80 °C.

Parabolic flight was conducted one afternoon in August 2024 at the Flight Research Laboratory, National Research Council of Canada, in Ottawa, using a modified Falcon-20 aircraft. Flight parameters were recorded at 10 Hz over a total duration of 1.3 h (of which 3,115 s was in the air). Parabolic maneuvers generated repeated periods of microgravity lasting approximately 18–20 s per parabola (0.0 ± 0.1 G; 1 G = 9.81 m s^−2^), each initiated and terminated by 18–20 s of ~2-G entry and exit phases. A total of nine parabolas were performed, yielding cumulative exposures of 166.9 s in microgravity and 386.8 s in hypergravity, with a variability of ±2.1 s across parabolas. The maximum altitude reached was 5888.9 m.

### Metagenomic shotgun sequencing

Vaginal swab and saliva samples were processed prior to DNA extraction. Vaginal swab samples were pulse-vortexed for 5 min at room temperature to release microbial cells into the transport medium, followed by centrifugation at 10,000 × g for 10 min at room temperature. The supernatant was carefully discarded, and the resulting pellet was resuspended in 500 μL of phosphate-buffered saline (PBS) for further processing. For saliva samples, 1 mL of sample was directly used for downstream processing. Subsequently, microbial DNA was isolated using the QIAamp DNA Microbiome Kit (Cat. No. 19092; Qiagen, Germany) according to the manufacturer’s instructions. The protocol involves selective removal of host DNA to enrich microbial DNA, followed by microbial cell lysis, protein digestion, and purification using silica membrane-based spin columns. The extracted DNA was eluted in 50 μL of elution buffer. DNA concentration and purity were initially assessed using a NanoDrop spectrophotometer (Thermo Fisher Scientific, United States). Accurate quantification was further performed using the Qubit fluorometer with the Qubit^™^ 1X dsDNA High Sensitivity (HS) Assay Kit (Cat. No. Q33230; Thermo Fisher Scientific, United States). Prior to library preparation, DNA samples were normalized based on Qubit-derived concentrations.

DNA quantification was performed using the Qubit dsDNA HS Assay Kit (Cat. No. Q32854; Thermo Fisher Scientific, United States) on a Qubit Fluorometer, following the manufacturer’s instructions. For library preparation, a total of 150 ng DNA in 30 μL was used. Sequencing libraries were prepared using the Illumina DNA Prep (M) Tagmentation Kit (Cat. No. 20060059; Illumina, Inc., 5200 Illumina Way, San Diego, CA, United States) according to the manufacturer’s protocol. During library preparation, each sample was barcoded with unique dual-index adapters using the Illumina DNA/RNA UD Indexes. The prepared libraries were purified using magnetic bead-based cleanup, and fragment size distribution was assessed prior to pooling. Libraries were normalized and pooled at equimolar concentrations for sequencing. The pooled libraries were sequenced using the NextSeq 1000/2000 P2 XLEAP-SBS Reagent Kit (300 cycles) (Cat. No. 20100985; Illumina, Inc., San Diego, CA, United States) on an Illumina NextSeq 1000 platform to generate paired-end reads (2 × 150 bp). Sequencing data were processed using the BCL Convert tool within the Illumina BaseSpace Sequence Hub to generate FASTQ files, which were subsequently downloaded for downstream bioinformatics analysis.

### Microbiome analysis

Metagenomic data were analyzed using established metagenomics pipelines. We used a phyton based wrapper called the KneadData tool^1^ to remove the host DNA and adapter sequences. First, Trimmomatic (Bolger et al., 2014) was used to remove the adapter sequences. Next, the raw sequences were aligned using Bowtie2 (Langmead and Salzberg, 2012) against a provided dataset containing the hg37 human assembly and human contamination sequences. Then, the cleaned sequences were taken and used for the taxonomic classification using the MetaPhlAn3.0 pipeline (Blanco-Míguez et al., 2023). The MetaPhlAn database consists of ~ 5.1 M unique clade-specific marker genes identified from ~1 M microbial genomes (~236,600 references and ~771,500 metagenomic assembled genomes), which allows unambiguous taxonomic assignments, an accurate estimation of organismal relative abundance, and species-level resolution for bacteria, archaea, eukaryotes, and viruses. Then all the generated outputs were merged using the *merge_metaphlan.py* script provided by MetaPhlAn. MetaPhlAn predicts the taxonomic composition of microbial communities by estimating the relative abundance of organisms (from phylum to species/strain level) using clade-specific marker genes. We also conducted pathway analysis which uncovers the metabolic potential of microorganisms within a sample. It integrates taxonomic and functional profiling to infer microbial metabolic activity. We have used the cleaned fastq files and identified potential pathways using HUMAnN 3.0 (HMP Unified Metabolic Analysis Network) (Beghini et al., 2021). HUMAnN 3.0 utilizes the ChocoPhlAn pangenome database for nucleotide-level alignment and the UniRef50 protein database for translated searches, enabling comprehensive identification of gene families and metabolic pathways. The resulting gene family abundances were normalized and regrouped into higher-level functional pathways, allowing quantitative estimation of pathway coverage and relative abundance. These pathway profiles were subsequently subjected to statistical analysis and correlated with bacterial species composition to explore functional differences and potential associations between microbial taxa and metabolic capabilities across samples.

The beta diversity (inter-community diversity) was calculated using the permutational multivariate analysis of variance (PERMANOVA) test. Alpha diversity (within sample diversity) analysis was calculated based on observe species, Shannon and Simpson index using the relative abundance obtained from the MetaPhlAn tool. Observed OTUs were calculated as the total number of unique taxa detected per sample, representing species richness, while community diversity was further assessed using the Shannon diversity index, which incorporates both species richness and evenness, and the Simpson diversity index, which estimates community dominance by measuring the probability that two randomly selected individuals belong to the same taxon; taxonomic composition and OTU estimation were derived using MetaPhlAn based on clade-specific marker genes. A Random Forest model using 500 trees were generated by using the MicrobiomeAnalyst platform to identify features discriminating between study groups (Lu et al., 2023). Given the limited sample size (*n* = 8 for saliva and vaginal swab), cross-validation approaches (e.g., k-fold validation) were not applied during model training, as such methods would not provide reliable estimates under these conditions. Instead, the analysis was conducted in an exploratory framework to identify features that may potentially discriminate between the studied groups. We acknowledge that the absence of cross-validation and the small sample size are important limitations.

Bacterium-pathway associations were inferred using Spearman’s rank correlation coefficient (*ρ*), a non-parametric measure selected due to the non-normal, compositional, and zero-inflated nature of microbiome abundance data. For each condition (pre- and postflight) and anatomical site (saliva and vaginal), pairwise Spearman correlations were computed between bacterial taxa abundance profiles and metabolic pathway abundance profiles across matched samples. Correlation analyses were performed independently within each dataset and site to avoid cross-condition or cross-site bias. Associations were considered robust if they met a predefined effect size threshold of |*ρ*| ≥ 0.4, indicating at least a moderate monotonic relationship. Statistical significance was assessed using two-tailed tests and resulting *p*-values were adjusted for multiple comparisons using the Benjamini–Hochberg false discovery rate (FDR) correction, with FDR <0.05 considered significant. The R scripts were used for correlation analysis is provided in Supplementary material. Sankey plots were generated to visualize the relationships between dominant microbial taxa and their associated metabolic pathways using the online tool SankeyMATIC.^2^ This approach enabled intuitive representation of the flow and relative contributions of bacterial species to specific functional pathways identified in the dataset. The width of the connecting bands in the Sankey diagram corresponds to the relative abundance or contribution of each taxon–pathway association, allowing clear identification of predominant microbial contributors and their functional roles.

### Saliva cortisol and proinflammatory cytokine analysis

Salivary cortisol and proinflammatory cytokines (TNF-*α*, IL-6, IL-8, IL-1β) were measured by Salimetrics (Carlsbad, CA). Samples were tested for salivary cortisol using a high-sensitivity enzyme immunoassay (Cat. No. 1-3002). Proinflammatory cytokines were measured by the electrochemiluminescence method; assay ranges were TNF-α: 0.04–1,572 pg/mL, IL-6: 0.06–2,912 pg/mL, IL-8: 0.07–2,488 pg/mL, IL-1β: 0.05–2,568 pg/mL. Data were assessed for normality using the Shapiro–Wilk test. The paired *t*-tests and Wilcoxon tests were applied to compare pre- and postflight samples. Significant taxa were identified using the paired sample *t*-test. GraphPad Prism v11 was used for plotting and statistical analysis.

### Urine short-chain fatty acids analysis

An aliquot of 1 mL urine was mixed with 10 mM sodium bicarbonate buffer, vortexed for 10 min, and sonicated for 10 min to extract soluble metabolites. Samples were centrifuged at 3,200 × g for 10 min, and 100 μL of the supernatant was subjected to liquid–liquid extraction by the addition of 1.0 mL tert-butyl methyl ether and 50 μL of 1.0 M HCl. After vortexing for 10 min and centrifugation at 12,900 × g for 10 min, ~700 μL of the organic phase was collected, syringe-filtered, and transferred to GC autosampler vials. All samples and standards were analyzed in duplicate by GC/MS or GC/FID. Short-chain fatty acids (SCFAs) were separated on a DB-FFAP capillary column (30 m × 0.32 mm × 0.5 μm; Agilent) using a GC-2023 system (Shimadzu) equipped with an AOC-30i auto-injector and operated with GC Real-Time Analysis software. The injector, transfer line, and ion source were maintained at 30 °C, 180 °C, and 220 °C, respectively. The oven temperature was programmed to 30 °C, then to 180 °C, and finally to 220 °C, with a 5 min hold. Mass spectra were acquired in electron impact mode at 70 eV (*m*/*z* 42–73; 0.2 s scan time). Acetate, propionate, butyrate, and valerate were identified by comparison with the National Institute of Standards and Technology (NIST) spectral library and confirmed using authentic standards. Quantification was performed in multiple reaction monitoring (MRM) mode, and concentrations were calculated from external calibration curves generated from serial dilutions of pure SCFA standards.

## Results

### Parabolic flight restructures the vaginal microbiome significantly compared to the oral microbiome

**Figure 1:**
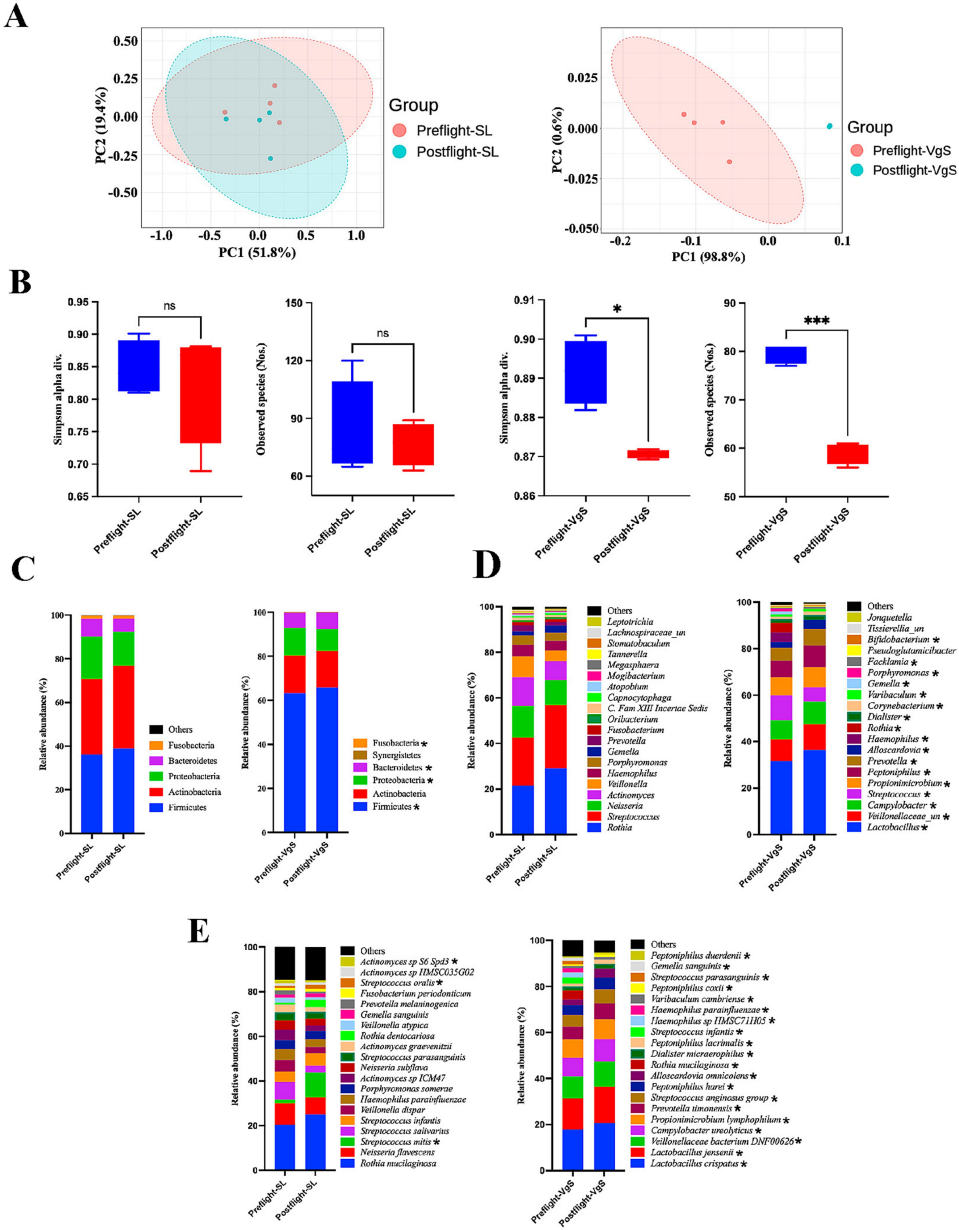
Diversity and taxonomy analysis for saliva and vaginal microbiome. **(A)** Beta diversity analysis: principal coordinates analysis (PCoA) plot of the Bray–Curtis dissimilarity showing beta-diversity of saliva (SL) and vaginal swab (VgS) microbiomes before and after parabolic flight. Pink (preflight) and teal (postflight) are colored by flight status. **(B)** Analysis of alpha-diversity in oral and vaginal microbiome, including observed species richness (Nos.) and Simpson alpha diversity, ^*^p < 0.05 and ^***^p < 0.001. **(C)** Phylum-level comparison of the relative abundance of bacterial communities in saliva and vaginal samples before and after parabolic flight. **(D)** Genus-level comparison of the relative abundance of bacterial communities in saliva and vaginal samples before and after parabolic flight. **(E)** Species-level comparison of the relative abundance of bacterial communities in saliva and vaginal samples before and after parabolic flight, ^*^p < 0.05.

To assess the impact of parabolic flight on oral and vaginal microbial communities, we compared pre- and postflight saliva (SL) and vaginal swab (VgS) microbiome data. Beta diversity was assessed using the Bray–Curtis dissimilarity index and visualized by principal coordinates analysis (PCoA). PCoA plot revealed partial separation between pre- and postflight oral microbiome samples, with substantial overlap between groups (Figure 1A). In contrast, the vaginal microbiome showed clear separation of microbiome between the pre- and postflight samples (Figure 1A). In terms of alpha diversity, saliva alpha diversity did not differ significantly between pre-and postflight samples, as shown in both observed species richness (number of observed species; NOS) and Simpson alpha diversity plots (Figure 1B). In contrast, vaginal microbiome samples had a significant decrease in both observed species richness (*p* < 0.0001) and Simpson alpha diversity (*p* < 0.05) following parabolic flight (Figure 1B).

**Figure 2:**
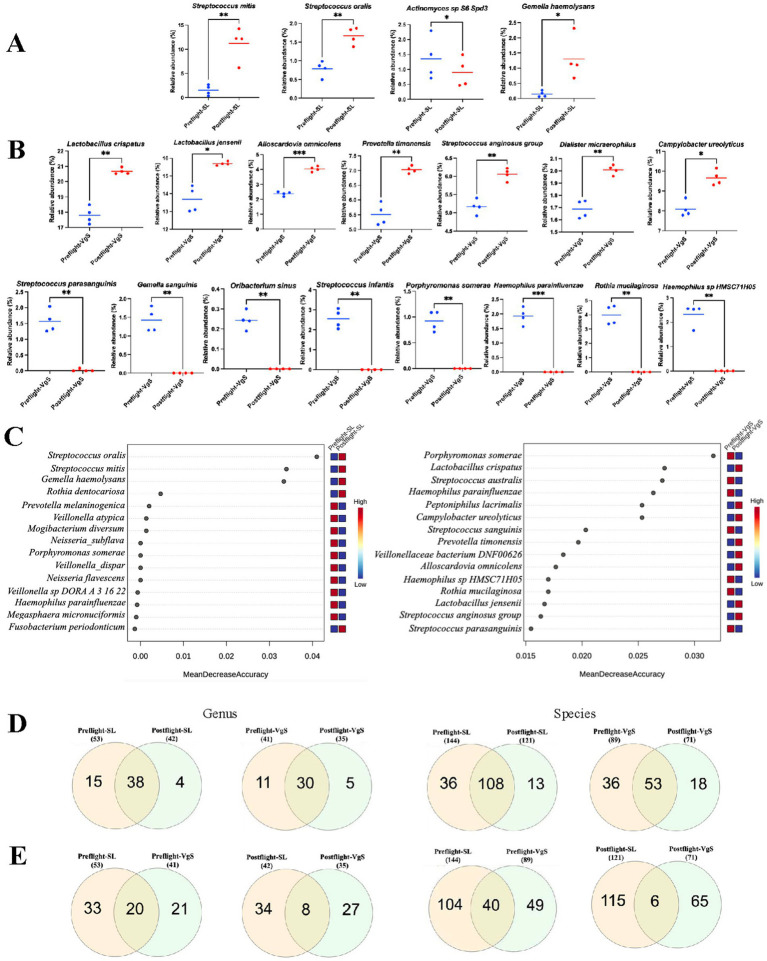
Species-level analysis. **(A)** Species-level relative abundance of selected oral bacterial taxa pre- and postflight comparison. **(B)** Species-level relative abundance of selected vaginal bacterial taxa pre- and postflight comparison, ^*^p < 0.05, ^**^p < 0.01, and ^***^p < 0.001. **(C)** Random Forest analysis of top 15 bacteria species to discriminate between pre- and postflight groups. Features are ranked by their contributions to classification accuracy (mean decrease accuracy). High (red): higher relative abundance, low (blue): lower relative abundance. **(D)** Unique genus and species for salivary and vaginal microbiome between pre- vs. postflight. **(E)** Unique genus and species between salivary and vaginal microbiome for pre- and postflight.

Phylum-level taxonomic profiling showed that saliva samples were dominated by *Firmicutes*, *Actinobacteria*, *Proteobacteria*, and *Bacteroidetes*, with no statistically significant changes in relative abundance between pre- and postflight (Figure 1C). In contrast, the vaginal microbiome showed significant phylum changes in postflight samples. The relative abundance of *Firmicutes* increased postflight (63.1% vs. 65.9% pre- vs. postflight, *p* = 0.0002), along with significant changes in the relative abundance of several other phyla (Figure 1C), highlighting restructuring at the phylum level. Similarly, at the genus level, the oral microbiome showed no statistically significant changes in relative abundance between pre- and postflight samples (Figure 1D). In contrast, the vaginal microbiome showed significant differences in several genera between pre- and postflight samples (Figure 1D). The relative abundance of *Lactobacillus* increased postflight (31.5% vs. 36.3%, pre- vs. postflight, *p* = 0.003), along with an increase in *Peptoniphilus* (7.14% vs. 9.28%, *p* = 0.0001), and *Alloscardovia* (2.37% vs. 4.04%, *p* = 0.0006). Postflight relative abundance decrease is also observed in several genera such as *Streptococcus, Haemophilus*, and *Rothia* (*p* < 0.05) (Figure 1D). A similar pattern was observed at the species level, with the oral microbiome remaining relatively stable between pre- and postflight samples compared to the vaginal microbiome. In the vaginal microbiome, significant postflight shifts are observed at the species level (Figure 1E). Within the oral microbiome, we have observed a limited number of statistically significant species such as *Streptococcus oralis* (0.78% vs. 1.66%, pre- vs. postflight, *p* = 0.004) and *Streptococcus mitis* (1.5% vs. 11.2%, *p* = 0.0009) in postflight (Figures 1E, 2A). In contrast, in the vaginal microbiome, a greater number of species showed significant changes in their relative abundance. The postflight relative abundance increased in both *Lactobacillus crispatus* (17.8% vs. 20.67%, *p* = 0.001) and *Lactobacillus Jensenii* (13.68% vs. 15.69%, *p* = 0.01) (Figures 1E, 2B). Postflight vaginal samples showed significantly increased relative abundance of other species, including *Alloscardovia omnicolens*, *Prevotella timonensis*, *Streptococcus anginosus* group, *Dialister microaerophilus*, and *Campylobacter ureolyticus* (Figure 2B), while *Haemophilus parainfluenzae*, *Haemophilus* sp. *HMSC71H05*, *Rothia mucilaginosa*, *Streptococcus infantis*, *Porphyromonas somerae*, *Gamella sanguinis*, and *Streptococcus parasanguinis* were significantly reduced postflight (Figure 2B). Overall, 2.54% of identified oral species showed statistically significant changes following flight, compared to 57.9% of identified vaginal species (*p* < 0.0001). In summary, parabolic flight was associated with significant restructuring of the vaginal microbiome but minimal changes in the oral microbiome, as demonstrated by beta diversity plots, reduced alpha diversity, and taxonomic redistribution (Figures 1A–E, 2A,B).

Random Forest classification model was used to identify the most important microbial species discriminating pre- and postflight responses (Figure 2C). In saliva samples, the top three ranked predictive species were *Streptococcus oralis*, *Streptococcus mitis*, and *Gemella haemolysans*, and each of them showed increased relative abundance postflight (Figure 2C). In the vaginal microbiome, *Porphyromonas somerae*, *Lactobacillus crispatus*, and *Streptococcus australis* emerged as the top ranked discriminative species. The increased abundance of *Lactobacillus crispatus*, along with decreased abundance of *Porphyromonas somerae* and *Streptococcus australis* were discriminative for postflight vaginal microbiome composition (Figure 2C).

Venn diagram used to identify shared and unique taxa between pre- and postflight samples in saliva and vaginal swabs (Figures 2D,E). At the genus level, saliva samples showed 38 genera shared between pre- and postflight conditions, with 15 and 4 genera unique to preflight and postflight, respectively. In vaginal samples, 30 genera were shared, while 11 and 5 genera were unique to preflight and postflight, respectively (Figure 2D). At the species level, 108 species were shared between pre- and postflight saliva samples, with 36 and 13 species unique to preflight and postflight, respectively (Figure 2D). In vaginal samples, 53 species were shared, with 36 and 18 species unique to pre- and postflight conditions, respectively (Figure 2D). Notably, the number of shared genera between saliva and vaginal samples decreased postflight, suggesting increased site-specific divergence following flight exposure (Figure 2E). The list of the unique and common genera and species are provided in Supplementary Table 1.

### Parabolic flight induces site-specific changes in microbiome associated pathways

**Figure 3:**
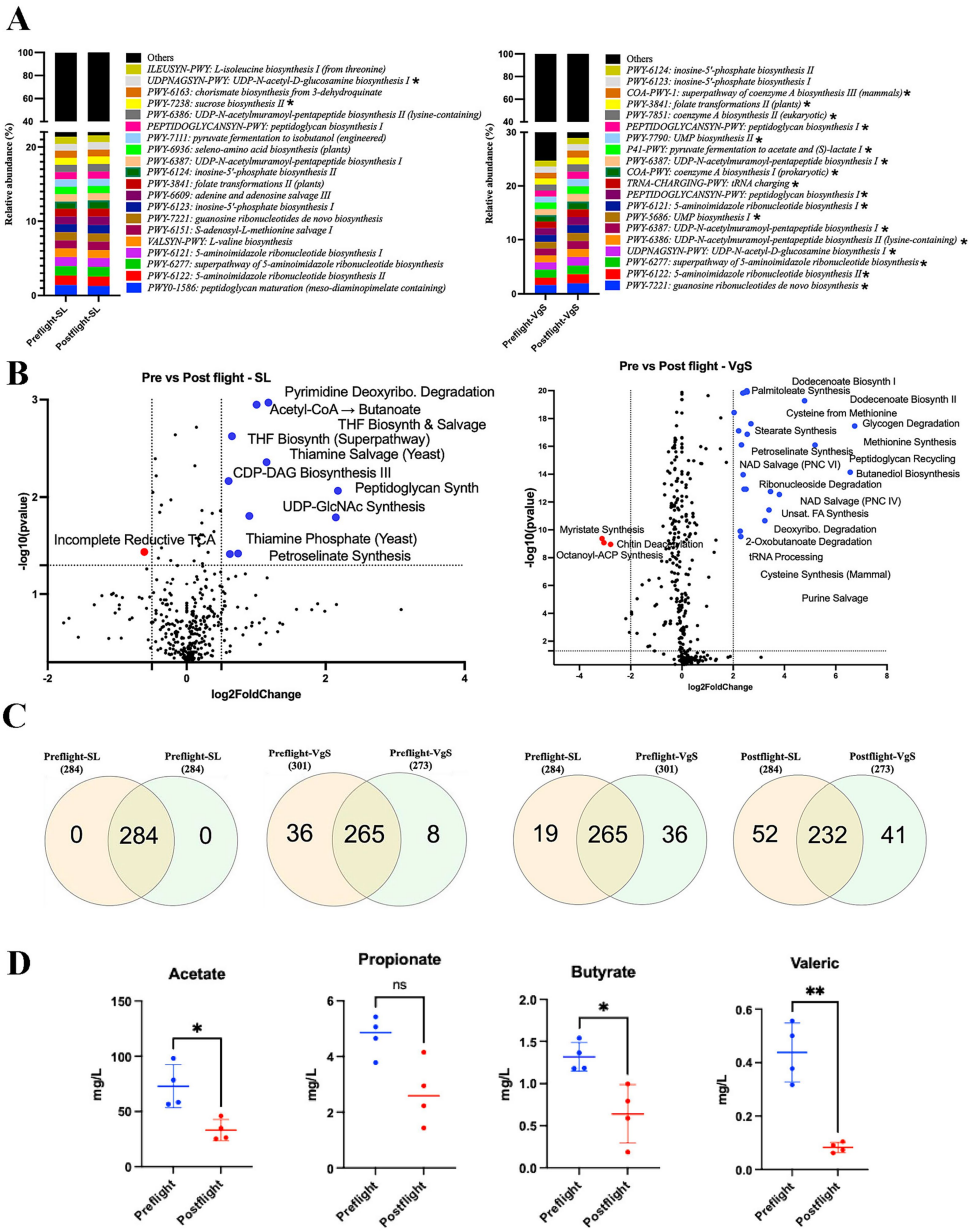
Pathways and urine SCFAs. **(A)** Top 20 microbial metabolic pathways shown in relative abundance (%) based on shotgun metagenomics pathway reconstruction using HuMANnN 3.0, ^*^p < 0.05. **(B)** Volcano plot for top pathways for salivary and vaginal microbiome. **(C)** Unique pathways between pre- and postflight and unique pathways between salivary and vaginal pathways. **(D)** Urine SCFAs, ^*^p < 0.05 and ^**^p < 0.01.

Metagenomic functional pathway abundances determined via HUMAnN 3.0 showed the top 20 microbial metabolic pathways contributing to the functional potential of saliva and vaginal microbiomes (Figure 3A). In saliva samples, pathway profiles were dominated by biosynthetic functions and showed minimal differences between pre- and postflight conditions. In contrast, vaginal microbiomes showed a greater redistribution of pathway abundances postflight, with multiple biosynthetic and energy pathways displaying significant differences between pre- and postflight, consistent with the observed taxonomic restructuring (Figure 3A). Further, the volcano plot depicted a greater number of significant enriched metabolic pathways in vaginal microbiome samples compared to oral microbiome samples (Figure 3B). In oral microbiome samples, 284 pathways were identified, and 58 of them showed significant change postflight (20.4%), whereas in vaginal samples, 309 pathways were identified and 190 of them (61.5%) were statistically significant (20.4% vs. 61.5%, *p* < 0.0001). In saliva samples, the top upregulated pathways in postflight were *PWY-5265: peptidoglycan biosynthesis II (staphylococci)* (Log2fc: 2.17, *p* = 0.008), *UDP-N-ACETYLGALSYN-PWY: UDP-N-acetyl-D-glucosamine biosynthesis II* (Log2fc: 2.14, *p* = 0.01), and *PWY-6612: superpathway of tetrahydrofolate biosynthesis* (Log2fc:1.17, *p* = 0.001). The top downregulated pathways were *PWY-6607: guanosine nucleotides degradation I* (Log2fc: −1.05, *p* = 0.01), and PWY*-6353: purine nucleotides degradation II (aerobic)* (Log2fc: −1.01, *p* = 0.01). In vaginal samples, the top upregulated pathways in postflight were *PWY-7388: octanoyl-ACP biosynthesis* (Log2fc: 3.11, *p* = 0.01), *PWY66-430: myristate biosynthesis (mitochondria)* (Log2fc:3.04, *p* = 0.02), and *PWY-7118: chitin deacetylation* (log2fc: 2.78, *p* = 0.02) (Figure 3B). The top downregulated metabolic pathways were *GLYCOCAT-PWY: glycogen degradation I* (Log2fc: −6.73, *p* = 0.0005), *PWY0-1261: anhydromuropeptides recycling I* (Log2fc: −6.56, *p* = 0.002), *PWY-7858: (5Z)-dodecenoate biosynthesis II* (Log2fc: −4.78, *p* = 0.0002). Among pathways significantly altered postflight, 33 were shared between oral and vaginal samples. Of these, 8 (24.2%) pathways changed in the same direction (either upregulated or downregulated) across both sites, whereas the remaining shared pathways (81.8%) showed opposite directions of change between oral and vaginal samples, indicating a site-specific functional response. We tried to identify the unique pathway between samples and within samples. In the case of saliva, we have not observed any unique pathway between pre- and postflight condition while vaginal swab showed 36 and 8 unique pathways for pre- and postflight condition. We have found several unique pathways between saliva and vaginal, which clearly demonstrate the sample heterogeneity (Figure 3C). The list of the unique and common pathways is provided in Supplementary Table 1.

Postflight urine short-chain fatty acids (SCFAs) were significantly decreased (acetate, butyrate, and valeric acid) (Figure 3D), indicating flight-specific SCFA profiles. The urine acetate levels were 72.83 ± 19.52 mg/L vs. 33.11 ± 9.55 mg/L (*p* = 0.01); urine butyrate levels were 1.31 ± 0.17 mg/L vs. 0.64 ± 0.34 mg/L (*p* = 0.04), and urine valeric acid levels were 0.43 ± 0.1 mg/L vs. 0.08 ± 0.01 mg/L pre- and postflight, respectively (Figure 3D).

**Figure 4:**
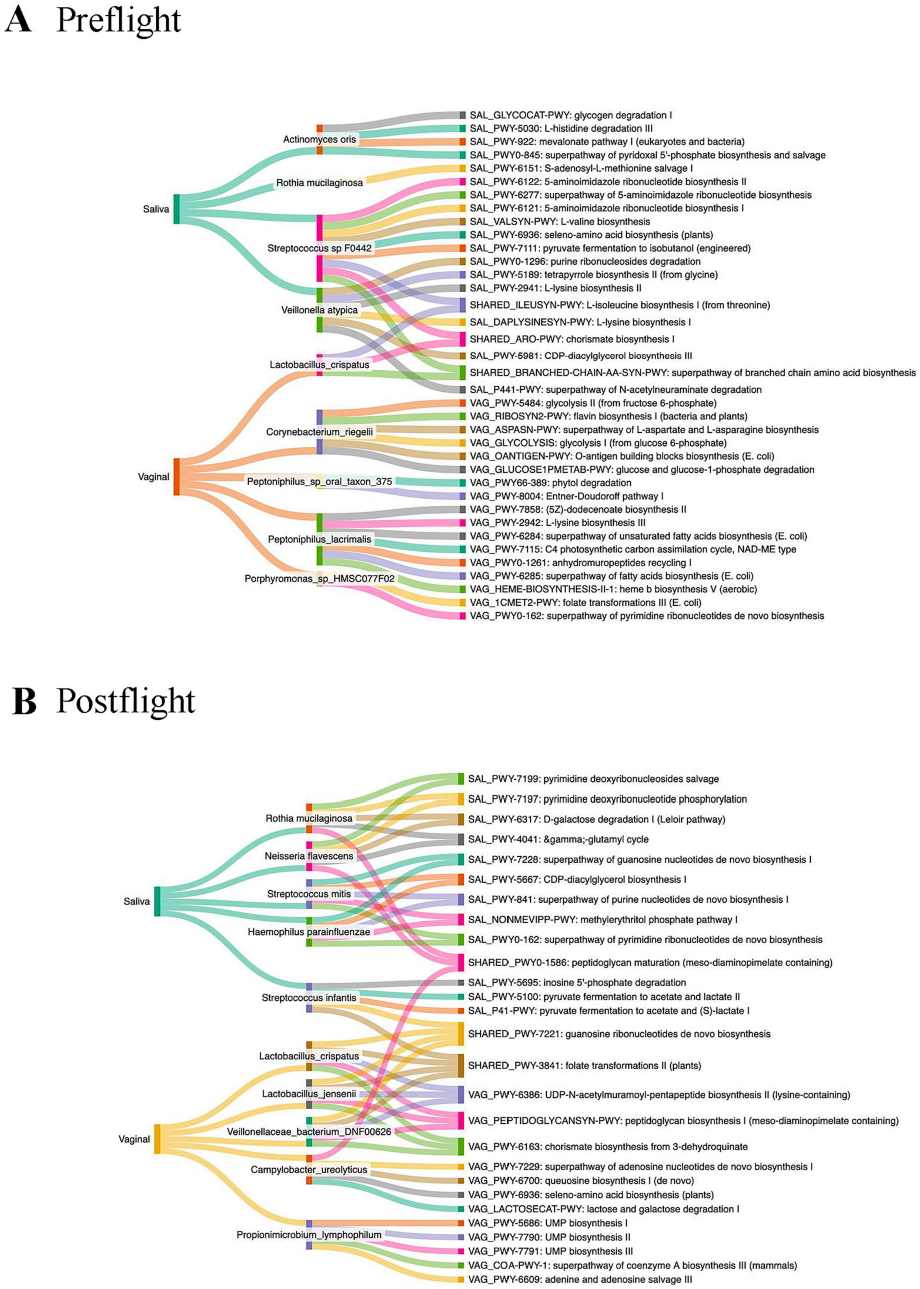
Sankey diagram for pathway analysis between saliva and vaginal species: **(A)** preflight and **(B)** postflight. Left nodes: body site, middle nodes: dominant species, right nodes: metabolic pathways.

The Sankey diagram showed the redistribution of preflight and postflight species for saliva and vaginal samples and associated pathways (Figures 4A,B). Vaginal samples exhibited greater redistribution of species, consistent with increased predominance of *Lactobacillus jensenii* postflight. There was an increased number of species-pathway connections observed postflight in both oral and vaginal samples (Figure 4B). In the pre-flight, we have seen three shared pathways (L-isoleucine biosynthesis, Chorismate biosynthesis I, and Superpathway of branched chain amino acid) between the saliva and vaginal microbiome. *Lactobacillus crispatus* (vaginal) and *Streptococcus* sp. *F0442* (saliva) contribute to all these three pathways. In case of postflight (Figure 4A). In postflight, Sankey network analysis revealed clear site-specific bacterium-pathway associations with a limited set of shared functional pathways across saliva and vaginal microbiomes. Salivary taxa, including *Rothia mucilaginosa*, *Neisseria flavescens*, *Streptococcus mitis*, and *Haemophilus parainfluenzae*, were predominantly linked to nucleotide metabolism, carbohydrate utilization, and fermentation-related pathways. In contrast, vaginal-associated bacteria such as *Lactobacillus crispatus*, *Lactobacillus jensenii*, *Veillonellaceae bacterium DNF00626*, and *Campylobacter ureolyticus* showed strong associations with peptidoglycan, chorismate, UMP, and coenzyme A biosynthesis pathways. Notably, shared pathways including peptidoglycan maturation, guanosine nucleotide biosynthesis, and folate transformations connected bacteria across both sites, indicating a conserved functional core despite site-specific metabolic specialization (Figure 4B).

### Parabolic flight is associated with host stress and microbiome interaction

**Figure 5:**
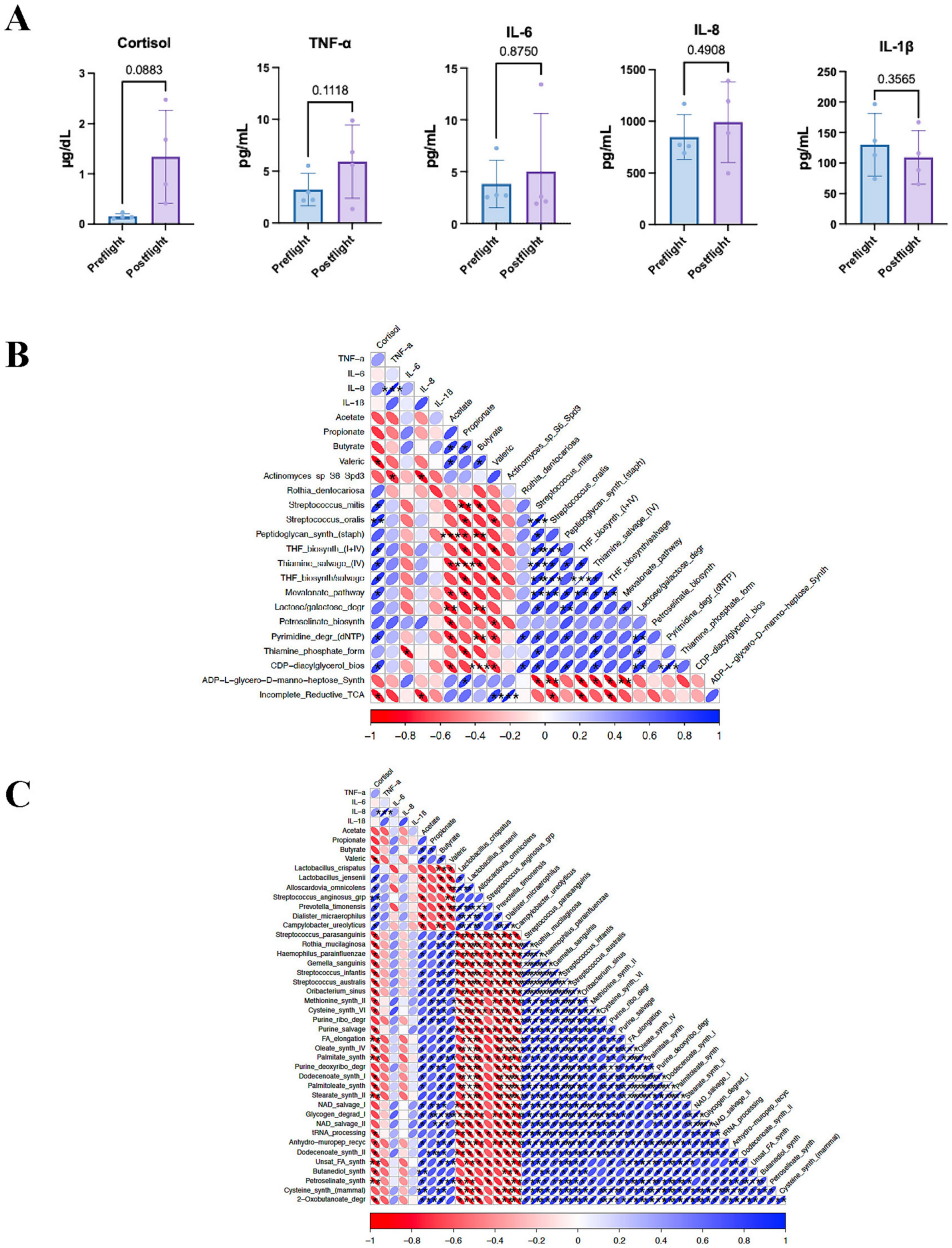
Salivary cortisol and proinflammatory cytokine levels and correlation analysis. **(A)** The mean salivary cortisol and proinflammatory cytokines levels between pre- and postflight samples. **(B)** Spearman correlation analysis of top species, pathways, metabolites, and inflammatory markers for oral microbiome. **(C)** Spearman correlation analysis of top species, pathways, metabolites, and inflammatory markers for vaginal microbiome. ^*^p < 0.05, ^**^p < 0.01, and ^***^p < 0.001.

To assess host physiological stress response to parabolic flight, salivary cortisol and proinflammatory cytokines were quantified before and after the flight (Figure 5A). Salivary cortisol levels increased postflight in all subjects, although the magnitude of increase varied between individuals. The mean saliva cortisol level was 0.15 ± 0.05 μg/dL vs. 1.34 ± 0.92 μg/dL pre- vs. postflight (*p* = 0.08) (Figure 5A). Proinflammatory cytokines exhibited heterogeneous responses, with overall increasing trends observed for TNF-*α*, IL-6, and IL-8 following flight and associated with intersubject variability. The mean saliva TNF-α level was 3.22 ± 1.57 pg/mL vs. 5.91 ± 3.53 pg/mL pre- vs. postflight (*p* = 0.11) (Figure 5A).

Correlation analysis incorporating top differentially abundant species, HUMAnN inferred metabolic pathways, urine SCFAs, salivary cortisol, and proinflammatory cytokines revealed modest but structured associations between host stress markers and the oral microbiome (Figure 5A). Salivary cortisol exhibited significant positive correlations with several core oral taxa, including *Streptococcus mitis* (*r* = 0.833, *p* = 0.01), and *Streptococcus oralis* (*r* = 0.857, *p* = 0.006). Cortisol levels were also positively correlated with multiple metabolic pathways, including tetrahydrofolate (THF) biosynthesis (I and V), thiamine salvage (IV), mevalonate pathway biosynthesis, and pyrimidine degradation (Figure 5B). In contrast, salivary cortisol had a negative correlation with urinary valeric acid concentrations (*r* = −0.833, *p* = 0.01) (Figure 5B). Notably, most metabolic pathways showed negative correlations with urinary SCFAs, suggesting an inverse relationship between microbial functional potential and systemic SCFA levels (Figure 5B). In the vaginal microbiome, correlation analysis revealed distinct associations between salivary cortisol levels and both species and metabolic pathways (Figure 5C). Cortisol levels were positively correlated with *Lactobacillus* species, with the strongest and statistically significant associations observed for *Lactobacillus jensenii* (*r* = 0.810, *p* = 0.01). In contrast, cortisol exhibited negative correlations with most microbial metabolic pathways. *Lactobacillus* species also showed negative correlations with the plotted pathways (*p* < 0.05) (Figure 5C). Overall, the vaginal microbiome showed greater number of correlations between metabolic pathways, cortisol, and cytokine levels (Figure 5C).

## Discussion

The vaginal microbiome is known to be a critical regulator of mucosal homeostasis, epithelial barrier integrity, and local immune function on Earth, yet it has not been explored in spaceflight research despite the increasing participation of women in human space exploration, and genitourinary pathologies being listed among top concerns in long-duration spaceflight (Blue et al., 2019). In asymptomatic women of reproductive age, human vaginal flora consists of a variety of aerobic and anaerobic species containing upwards of 10^10–11^ bacteria (Chen et al., 2017). The vaginal microbiome has a dynamic ecosystem, with predominant taxa including *Lactobacillus, Peptococcus, Bacteroides, Staphylococcus*, and *Corynebacterium* species. *Lactobacilli* constitute 70% of the vaginal flora, distinguishing the human vaginal microbiome from other mammals (Bartlett et al., 1977; Miller et al., 2016). *Lactobacilli* play a protective role, inhibiting the growth of pathogens by maintaining the vaginal pH between 3.5 and 4.5 through the production of lactic acid, hydrogen peroxide, fatty acids, and other organic acids (Ogata et al., 1986). Common *Lactobacillus* organisms populating the vaginal microbiome include *L. crispatus, L. gasseri, L. iners,* and *L. jensenii* (de Souza et al., 2023). The vaginal microbiome has a regulated homeostatic ecosystem, and vaginal dysbiosis is associated with increased risk of infections, including bacterial vaginosis (BV), vulvovaginal candidiasis, and sexually transmitted infections (Chee et al., 2020). Beyond infections, vaginal dysbiosis has also been linked to adverse reproductive outcomes such as preterm birth, gynecologic diseases, gynecologic cancers, and postoperative surgical site infections (Han et al., 2021; Javadi et al., 2024; Soper, 2020).

Spaceflight stressors, including altered gravity and psychosocial stress, are known to dysregulate immune function and perturbate host microbe interactions, potentially increasing susceptibility to opportunistic infections and impairing host defense mechanisms (Winer et al., 2025). Both spaceflight and spaceflight analog environments, such as simulated microgravity platforms, reported measurable changes in the human microbiome (Liu et al., 2020; Ramos-Nascimento et al., 2023). Furthermore, recent studies showed that even short duration of spaceflight evokes site-specific microbiome changes across multiple body sites, highlighting that microbiome communities can respond differently depending on the site and environmental exposure (Tierney et al., 2024). Building on these data, our study expands space medicine microbiome research to include the female-specific microbiome. Consistent with the body site specific context, beta- and alpha diversity analysis showed that the oral microbiome had only modest changes following parabolic flight, with substantial overlap in community composition and no significant alterations in diversity metrics (Figures 1, 2). In contrast, the vaginal microbiome displayed significant postflight changes in both beta- and alpha-diversity, reflecting marked restructuring of the community and reduced microbial diversity. These differences likely reflect intrinsic ecological distinctions between these two sites. Compared to the oral microbiome, the vaginal microbiome has lower diversity, is more tightly regulated by hormones, and has less exposure to the external environment (Holdcroft et al., 2023; Human Microbiome Project Consortium, 2012). Such features may cause the vaginal microbiome to be more sensitive to acute physiological changes associated with altered gravity and stress.

Taxonomic profiling showed that vaginal microbiome restructuring was characterized by increased dominance of *Firmicutes*, particularly *Lactobacillus* species, accompanied by redistribution and depletion of some anaerobic taxa (Figure 2B). Species-level results showed that *Lactobacillus crispatus* and *Lactobacillus jensenii* are key contributors to the microbiome community, with increased relative abundance postflight (Figure 2B). Random Forest model analysis further highlighted *Lactobacillus crispatus* as a key discriminator between pre- and postflight samples, reflecting its increased relative abundance postflight (Figure 2C). While *Lactobacillus* dominance reflects the healthy vaginal microbiome in women of reproductive age, the concurrent reduction in microbial diversity observed here may also reflect a stress response associated with microbiota restructuring. Further metagenomic pathway analysis demonstrated that vaginal microbiome changes were accompanied by shifts in metabolic pathways. Postflight vaginal samples showed enrichment of pathways related to fatty acid biosynthesis, nucleotide salvage and biosynthesis, amino acid metabolism, and peptidoglycan recycling (Figures 3A,B). These results suggest that the vaginal microbiome responds to parabolic flight not only through microbiome community remodeling but also through functional metabolic remodeling. Parabolic flight is characterized by alternating periods of hypergravity and microgravity. Simulated microgravity experiments showed that probiotic bacteria, *Lactobacillus acidophilus*, altered growth kinetics, and decreased sensitivity of bacteria to certain antibiotics, despite no major changes in their morphology (Shao et al., 2017). These findings support the concept that *Lactobacillus* species may adapt functionally to altered gravity environments.

Parabolic flight also resulted in host stress responses, reflected by increased salivary cortisol levels across all participants, with interindividual variability (Figure 5A). This finding is consistent with prior studies showing that parabolic flight and spaceflight environments activate neuroendocrine and immune stress pathways, as reflected by changes in cortisol and inflammatory cytokines (Krieger et al., 2021; Schneider et al., 2007). Stress has been shown to influence both oral and vaginal microbiomes in terrestrial contexts (Amabebe and Anumba, 2018; Charalambous et al., 2024), supporting the biological plausibility of stress and microbiome association under altered gravity conditions. Correlation analyses are further consistent with site specific response and revealed distinct patterns of interaction across anatomical sites. In the oral microbiome, associations between cortisol, cytokines, microbial taxa, and metabolic pathways were modest (Figure 5B). In contrast, the vaginal microbiome exhibited a more significant number of correlations between cortisol, dominant *Lactobacillus* species, metabolic pathways, and urinary valeric acid. Importantly, negative correlations between *Lactobacillus* dominance and multiple metabolic pathways likely reflect functional change rather than diminished microbial activity (Figure 5C).

The strengths of this study include exploration of an understudied area, the female reproductive tract microbiome within the context of space medicine, the use of paired pre- and postflight sampling, metagenomic shotgun sequencing for microbiome analysis, and integration of different analytical approaches (i.e., Random Forest, Sankey diagram, correlograms) to provide a robust and internally consistent assessment of microbiome responses. A key limitation of this study is its focus on parabolic flight, which may not fully capture the biological effects of long duration spaceflight, as well as the relatively small sample size which is common in space medicine. Further, it would be interesting to study the duration and permanence of the shift in vaginal flora, and its incidence with genitourinary pathology in follow-on studies, particularly in long-duration spaceflight. While the short duration of microgravity exposure limits some extrapolation to long-duration missions, the paired within-subject design reduces individual variability and supports the detection of acute shifts. Our results are primarily attributed to gravity effects and host stress, but other factors need to be considered. For example, the aircraft cabin conditions (i.e., temperature, relative humidity), flight suits, motion sickness and hydration. Further, owing to the nature of the parabolic flight profile, it is impossible to determine whether the changes seen reflect exposure to hypergravity, reduced gravity, or both, and follow-on studies might benefit from isolating these effects.

In conclusion, we demonstrated that parabolic flight induces body site-specific microbiome responses in women of reproductive age, with the vaginal microbiome showing robust taxonomic, and associated metabolic pathway changes, as compared to the oral microbiome. These findings suggest that the vaginal microbiome may exhibit a greater sensitivity to acute physiological stressors and altered gravity exposure. Given the critical role of the vaginal microbiome in mucosal immunity, infection susceptibility, and reproductive health, these results have direct relevance for risk assessment and countermeasure development for female crew members. Our work serves as a first step in understanding correlations in parabolic flight ahead of implementing the same research in suborbital and longer-duration of orbital flight. As human space exploration advances, these findings underscore the importance of incorporating female-specific microbiome research into space medicine to support risk assessment and countermeasure development. Future studies incorporating larger cohorts, suborbital and orbital missions will be critical to extend these findings.

## Data Availability

The datasets presented in this study can be found in online repositories. The names of the repository/repositories and accession number(s) can be found at: https://www.ncbi.nlm.nih.gov/, PRJNA1424208.

## Footnotes

1
https://github.com/biobakery/kneaddata

2
https://sankeymatic.com/build/

## Supplementary material

SUPPLEMENTARY TABLE 1 The list of unique and common genus, species and pathways between pre- and postflight conditions.

SUPPLEMENTARY MATERIAL R scripts.
