# Supplementary material for: Parabolic flight induces site specific microbiome changes in women
Source: Front Microbiol. 2026 May 25;17:1817099. doi: 10.3389/fmicb.2026.1817099 (PMC13243427; doi:10.3389/fmicb.2026.1817099)
Supplement: SUPPLEMENTARY TABLE 1 — The list of unique and common genus, species and pathways between pre- and postflight conditions. [file Table_1.DOCX]

##Supplementary document: Correlation script

#### Main correlation + corrplot + Excel export (with significance highlighting in BOTH sheets)

# Load libraries

library(tidyverse)

library(corrplot)

library(RColorBrewer)

library(Hmisc) # rcorr (correlation + p-values)

library(openxlsx) # Excel export + formatting

# 1. Read data

x <- read.csv(

"Str-gene-immune.csv", #Change the file name here

check.names = FALSE

)

# 2. Convert to numeric matrix

y <- as.matrix(x)

# 3. Compute correlation + significance (Spearman)

corr_res <- rcorr(y, type = "spearman")

corr <- corr_res$r # correlation matrix

pval <- corr_res$P # p-value matrix

# 4. Define color palette (red = negative, blue = positive)

col <- colorRampPalette(c("red", "white", "blue"))(200)

# -------------------------------

# EXPORT TO EXCEL (r + p + stars) WITH SIGNIFICANCE HIGHLIGHTING

# -------------------------------

sig_cutoff <- 0.05 # highlight if p < 0.05 (change if needed)

# Convert to data frames for writing

corr_df <- as.data.frame(corr)

pval_df <- as.data.frame(pval)

# Create stars matrix

stars <- matrix("", nrow = nrow(pval), ncol = ncol(pval), dimnames = dimnames(pval))

stars[pval < 0.05] <- "*"

stars[pval < 0.01] <- "**"

stars[pval < 0.001] <- "***"

# Workbook

wb <- createWorkbook()

# Sheet 1: Spearman r

addWorksheet(wb, "Spearman_r")

writeData(wb, "Spearman_r", cbind(Variable = rownames(corr_df), corr_df), rowNames = FALSE)

# Sheet 2: P-values

addWorksheet(wb, "P_values")

writeData(wb, "P_values", cbind(Variable = rownames(pval_df), pval_df), rowNames = FALSE)

# Sheet 3: significance stars

addWorksheet(wb, "Signif_stars")

writeData(wb, "Signif_stars", cbind(Variable = rownames(stars), as.data.frame(stars)), rowNames = FALSE)

# Dimensions

n <- nrow(corr)

m <- ncol(corr)

data_rows <- 2:(n + 1) # row 1 is header

data_cols <- 2:(m + 1) # col 1 is "Variable"

# Styles

sigStyle_r <- createStyle(

textDecoration = "bold",

fgFill = "#FFF2CC" # light yellow for significant r cells

)

sigStyle_p <- createStyle(

textDecoration = "bold",

fgFill = "#C6EFCE" # light green for significant p cells

)

# Apply number formatting (avoid scientific notation like 3.518E-01)

addStyle(

wb, "Spearman_r",

createStyle(numFmt = "0.000"),

rows = data_rows,

cols = data_cols,

gridExpand = TRUE,

stack = TRUE

)

addStyle(

wb, "P_values",

createStyle(numFmt = "0.000000"), # show small p-values without E-notation

rows = data_rows,

cols = data_cols,

gridExpand = TRUE,

stack = TRUE

)

# Find significant cells (skip diagonal)

sig_idx <- which(pval < sig_cutoff, arr.ind = TRUE)

sig_idx <- sig_idx[sig_idx[,1] != sig_idx[,2], , drop = FALSE]

# Highlight significant cells in Spearman_r sheet

for (k in seq_len(nrow(sig_idx))) {

i <- sig_idx[k, 1]

j <- sig_idx[k, 2]

addStyle(

wb, "Spearman_r", sigStyle_r,

rows = i + 1, # +1 header row

cols = j + 1, # +1 because first column is Variable

gridExpand = FALSE,

stack = TRUE

)

}

# Highlight significant cells in P_values sheet

for (k in seq_len(nrow(sig_idx))) {

i <- sig_idx[k, 1]

j <- sig_idx[k, 2]

addStyle(

wb, "P_values", sigStyle_p,

rows = i + 1,

cols = j + 1,

gridExpand = FALSE,

stack = TRUE

)

}

# Save Excel

saveWorkbook(

wb,

file = "Str-gene-immune_out.xls",

overwrite = TRUE

)

# -------------------------------

# CORRPLOT FIGURE (as you had it)

# -------------------------------

par(mar = c(1, 1, 3, 1))

corrplot(

corr,

method = "ellipse",

type = "lower",

col = col,

bg = "white",

tl.col = "black",

tl.srt = 45,

tl.cex = 0.7,

outline = FALSE,

addCoef.col = NULL,

p.mat = pval,

sig.level = c(0.001, 0.01, 0.05),

insig = "label_sig",

pch.cex = 1.0,

pch.col = "black",

diag = FALSE

)
