# Supplementary material for: Parabolic flight induces site specific microbiome changes in women
Source: Front Microbiol. 2026 May 25;17:1817099. doi: 10.3389/fmicb.2026.1817099 (PMC13243427; doi:10.3389/fmicb.2026.1817099)
Supplement: Supplementary file 3 [file Data_Sheet_1.PDF]

	Event	Elapsed Time (s) or Time (s)	Uncertainty (s)
**Parabola 1**	2-G Climb Start	1209.5	1.5
	Push over Start	1222.2	1.5
	2-G Climb Duration	12.7	2.1
	0G Start	1232.0	1.5
	0G End	1250.7	1.5
	0G Duration	18.7	2.1
	2-G Recovery End	1270.3	1.5
	2-G Recovery Duration	19.6	2.1
	Parabola Duration	51.0	2.1
**Parabola 2**	2-G Climb Start	1274.5	1.5
	Push over Start	1292.4	1.5
	2-G Climb Duration	17.9	2.1
	0G Start	1300.0	1.5
	0G End	1317.2	1.5
	0G Duration	17.2	2.1
	2-G Recovery End	1336.8	1.5
	2-G Recovery Duration	19.6	2.1
	Parabola Duration	54.7	2.1
**Parabola 3**	2-G Climb Start	1348.4	1.5
	Push over Start	1367.3	1.5
	2-G Climb Duration	18.9	2.1
	0G Start	1375.4	1.5
	0G End	1392.7	1.5
	0G Duration	17.3	2.1
	2-G Recovery End	1416.0	1.5
	2-G Recovery Duration	23.3	2.1
	Parabola Duration	59.5	2.1
**Parabola 4**	2-G Climb Start	1429.8	1.5
	Push over Start	1450.7	1.5
	2-G Climb Duration	20.9	2.1
	0G Start	1459.7	1.5
	0G End	1477.6	1.5
	0G Duration	17.9	2.1
	2-G Recovery End	1506.6	1.5
	2-G Recovery Duration	29.0	2.1
	Parabola Duration	67.8	2.1
**Parabola 5**	2-G Climb Start	1531.3	1.5
	Push over Start	1551.4	1.5
	2-G Climb Duration	20.1	2.1
	0G Start	1558.5	1.5
	0G End	1576.3	1.5
	0G Duration	17.8	2.1
	2-G Recovery End	1604.6	1.5
	2-G Recovery Duration	28.3	2.1
	Parabola Duration	66.2	2.1
**Parabola 6**	2-G Climb Start	1987.1	1.5
	Push over Start	2008.8	1.5
	2-G Climb Duration	21.7	2.1
	0G Start	2013.3	1.5
	0G End	2032.6	1.5
	0G Duration	19.3	2.1
	2-G Recovery End	2048.8	1.5
	2-G Recovery Duration	16.2	2.1
	Parabola Duration	57.2	2.1
**Parabola 7**	2-G Climb Start	2069.5	1.5
	Push over Start	2089.2	1.5
	2-G Climb Duration	19.7	2.1
	0G Start	2094.8	1.5
	0G End	2113.9	1.5
	0G Duration	19.1	2.1
	2-G Recovery End	2146.2	1.5
	2-G Recovery Duration	32.3	2.1
	Parabola Duration	71.1	2.1
**Parabola 8**	2-G Climb Start	2394.4	1.5
	Push over Start	2415.5	1.5
	2-G Climb Duration	21.1	2.1
	0G Start	2420.5	1.5
	0G End	2440.3	1.5
	0G Duration	19.8	2.1
	2-G Recovery End	2459.0	1.5
	2-G Recovery Duration	18.7	2.1
	Parabola Duration	59.6	2.1
**Parabola 9**	2-G Climb Start	2461.9	1.5
	Push over Start	2480.7	1.5
	2-G Climb Duration	18.8	2.1
	0G Start	2487.8	1.5
	0G End	2507.6	1.5
	0G Duration	19.8	2.1
	2-G Recovery End	2535.6	1.5
	2-G Recovery Duration	28.0	2.1
	Parabola Duration	66.6	2.1

Event	Elasped Time (s) or Time (s)	Uncertainty (s)
Takeoff	570.0	1.5
Landing	3685.0	1.5
Flight time	3115.0	2.1
Total Time in 2G Climb	171.8	4.5
Total Time in 2G Recovery	215.0	4.5
Total Time in 0-G	166.9	4.5

Max Altitude (m) 5888.9

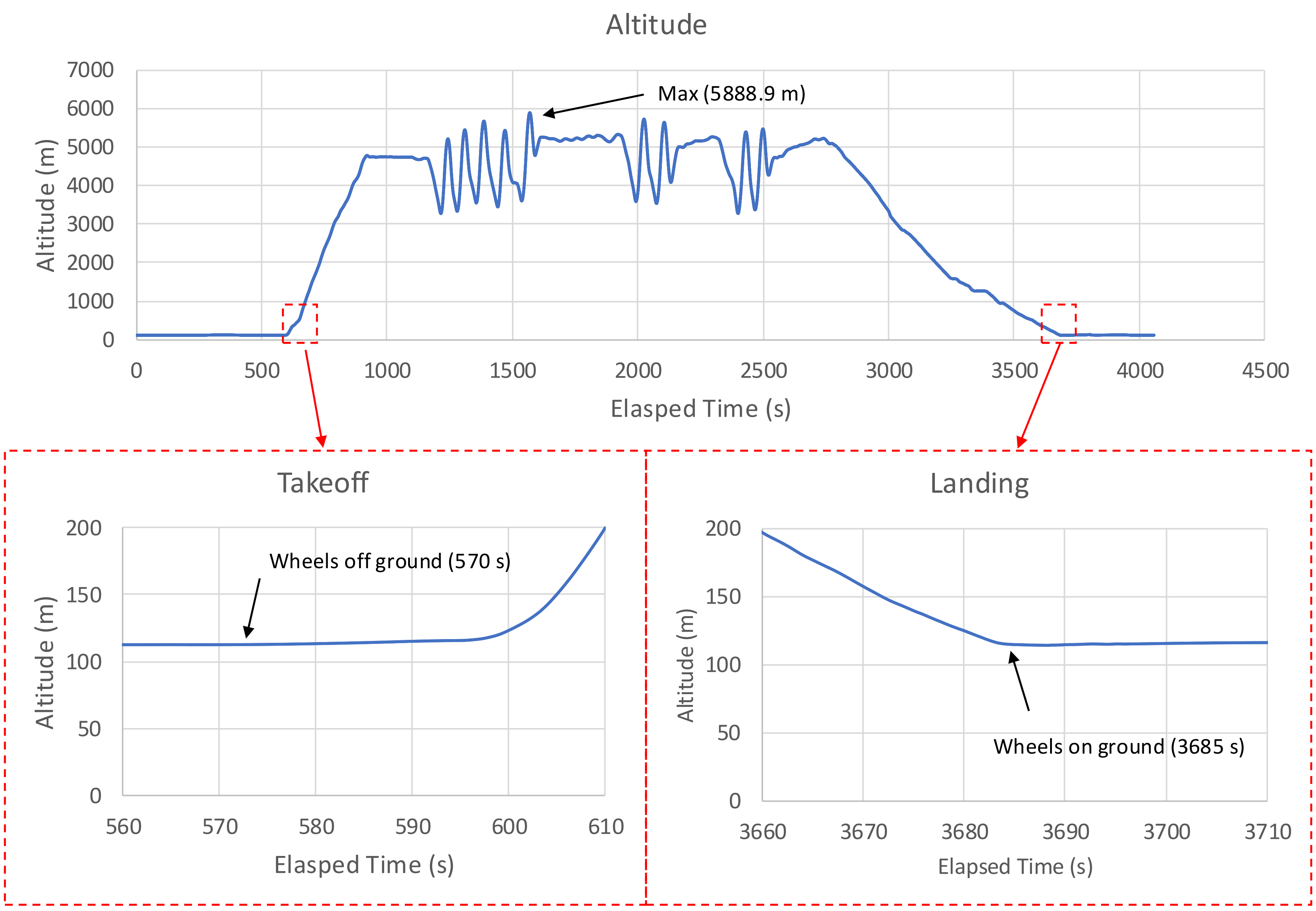

Altitude
Max (5888.9 m)
Altitude (m)
Elasped Time (s)
Takeoff
Wheels off ground (570 s)
Altitude (m)
Elasped Time (s)
Landing
Wheels on ground (3685 s)
Altitude (m)
Elapsed Time (s)

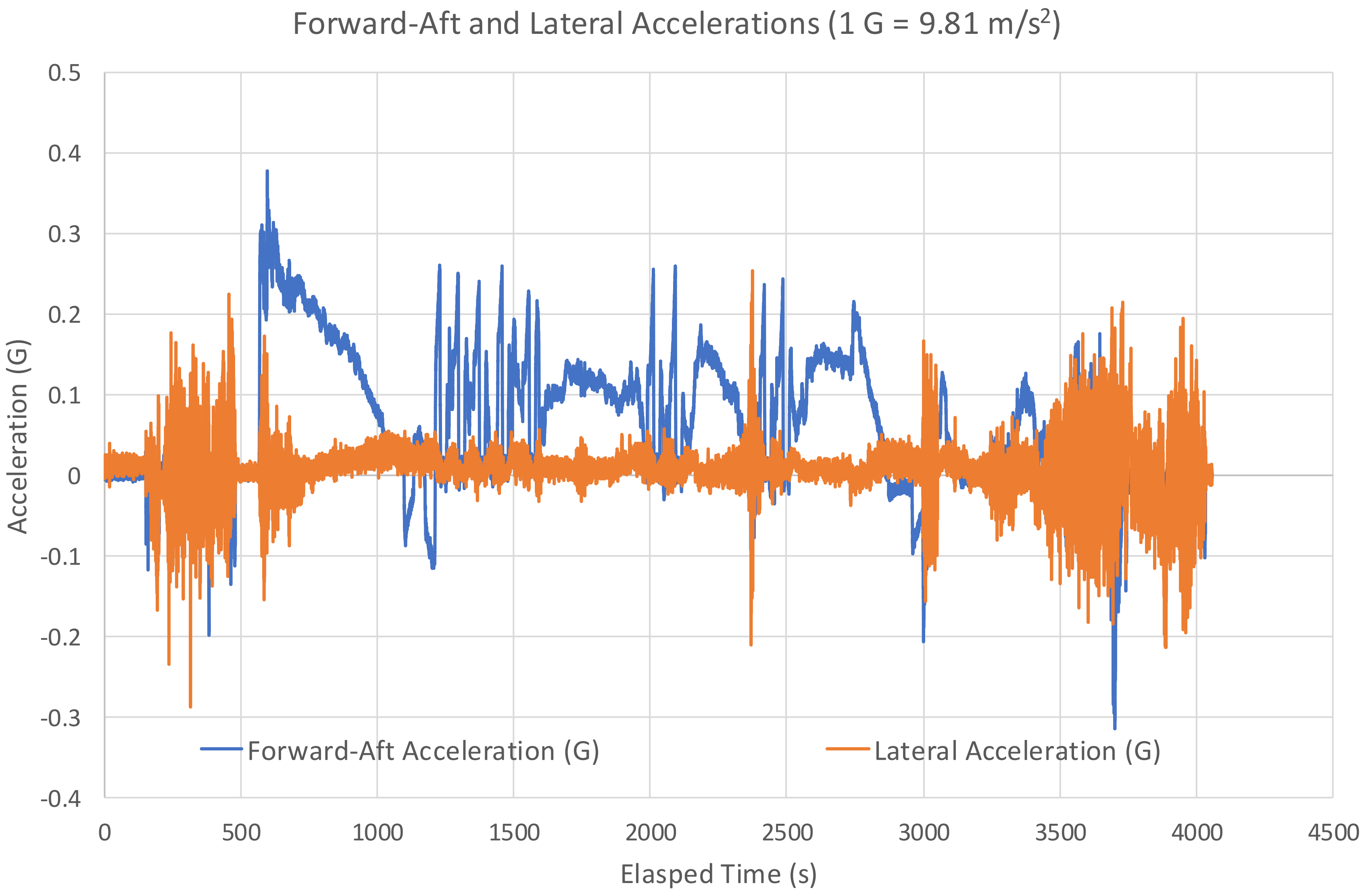

Forward-Aft and Lateral Accelerations (1 G = 9.81 m/s2)
Acceleration (G)
Forward-Aft Acceleration (G)
Lateral Acceleration (G)
Elasped Time (s)

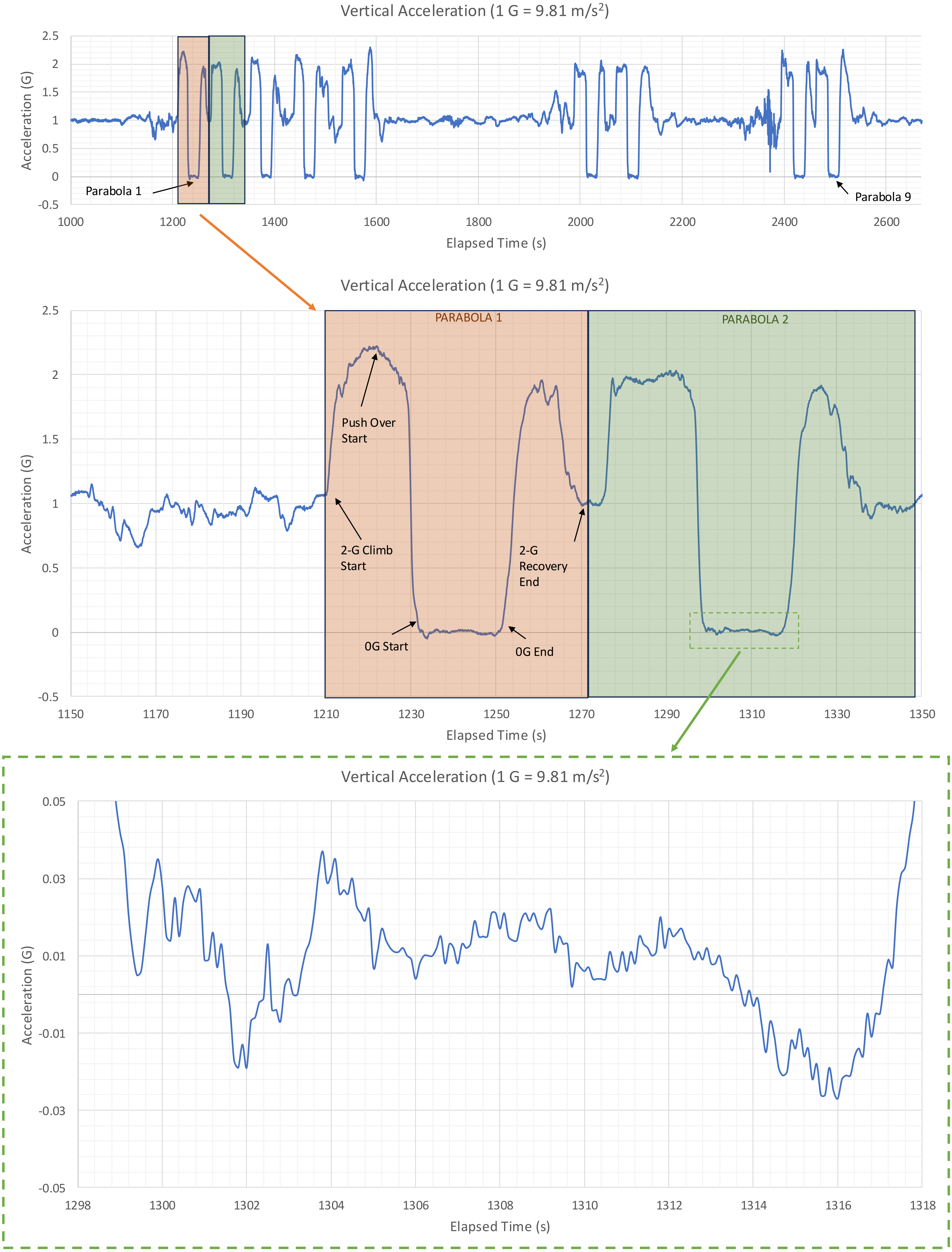
Vertical Acceleration (1 G = 9.81 m/s²)
Acceleration (G)
Elapsed Time (s)
Parabola 1
Parabola 9
Vertical Acceleration (1 G = 9.81 m/s²)
PARABOLA 1
PARABOLA 2
Push Over Start
2-G Climb Start
2-G Recovery End
0G Start
0G End
Acceleration (G)
Elapsed Time (s)
Vertical Acceleration (1 G = 9.81 m/s²)
Acceleration (G)
Elapsed Time (s)
